# Lys169 of Human Glucokinase Is a Determinant for Glucose Phosphorylation: Implication for the Atomic Mechanism of Glucokinase Catalysis

**DOI:** 10.1371/journal.pone.0006304

**Published:** 2009-07-20

**Authors:** Jian Zhang, Chenjing Li, Ting Shi, Kaixian Chen, Xu Shen, Hualiang Jiang

**Affiliations:** 1 Center for Drug Discovery and Design, State Key Laboratory of Drug Research, Shanghai Institute of Materia Medica, Shanghai Institutes for Biological Sciences, Chinese Academy of Sciences, and Graduate School of Chinese Academy of Sciences, Shanghai, China; 2 School of Pharmacy, East China University of Science and Technology, Shanghai, China; University of Canterbury, New Zealand

## Abstract

Glucokinase (GK), a glucose sensor, maintains plasma glucose homeostasis via phosphorylation of glucose and is a potential therapeutic target for treating maturity-onset diabetes of the young (MODY) and persistent hyperinsulinemic hypoglycemia of infancy (PHHI). To characterize the catalytic mechanism of glucose phosphorylation by GK, we combined molecular modeling, molecular dynamics (MD) simulations, quantum mechanics/molecular mechanics (QM/MM) calculations, experimental mutagenesis and enzymatic kinetic analysis on both wild-type and mutated GK. Our three-dimensional (3D) model of the GK-Mg^2+^-ATP-glucose (GMAG) complex, is in agreement with a large number of mutagenesis data, and elucidates atomic information of the catalytic site in GK for glucose phosphorylation. A 10-ns MD simulation of the GMAG complex revealed that Lys169 plays a dominant role in glucose phosphorylation. This prediction was verified by experimental mutagenesis of GK (K169A) and enzymatic kinetic analyses of glucose phosphorylation. QM/MM calculations were further used to study the role of Lys169 in the catalytic mechanism of the glucose phosphorylation and we found that Lys169 enhances the binding of GK with both ATP and glucose by serving as a bridge between ATP and glucose. More importantly, Lys169 directly participates in the glucose phosphorylation as a general acid catalyst. Our findings provide mechanistic details of glucose phorphorylation catalyzed by GK, and are important for understanding the pathogenic mechanism of MODY.

## Introduction

Glucokinase (GK) is a glycolic enzyme that catalyzes the phosphorylation of glucose to glucose-6-phosphate in the first step of glycolysis. Expressed in the liver, pancreas, brain and gut, GK plays a key role in maintaining glucose homeostasis through regulation of glucose-dependent insulin secretion in pancreatic cells, and glucose uptake and storage in the liver [1]–[3]. Clinical evidences have shown that GK might be an important therapeutic target for treating metabolic diseases such as maturity-onset diabetes of the young (MODY) and persistent hyperinsulinemic hypoglycemia of infancy (PHHI) [4]–[7].

Although GK belongs to the hexokinase family [8], GK is distinctive from other hexokinases because of its relatively low glucose affinity in the range of blood-glucose levels and its positive cooperative kinetics. These properties allow GK to rapidly respond to changes in glucose concentrations under physiological conditions, and thereby function as a glucose sensor [9]. Various studies have demonstrated that GK is a monomeric enzyme whose allosteric mechanism is different from those found in other extensively studied oligomeric allosteric enzymes [10]–[14]. Comparison of the crystal structures of GK in closed state (active state) and super-open state (inactive state) shows that GK may undergo a global conformational change between these two states, and such a conformational change may be responsible for the special allosteric characteristics of GK [15]. Recently, we used molecular dynamics simulation method to show that the global conformational transition pathway between the two states of GK includes three intermediate steps, identified by free energy scanning of snapshots throughout the pathway. The computational predictions were verified by mutagenesis and enzymatic kinetic analysis [16]. These studies gave the underlying principle of the enzymatic mechanism of GK and provided the explanations for the sigmoidal kinetic effect and the mnemonical mechanism for the cooperativity of GK with respect to glucose phosphorylation [17].

Recently, data for over 100 different mutations in the GK genes, potentially contributing to the development of an autosomal dominant form of type 2 diabetes, have been collected [18]. Among the data, some mutations in the MODY phenotype were found to be linked to alterations in both GK kinetics and regulation activities [19]. The catalytic mechanism of GK holds the most critical information for understanding the relation between the mutations in the MODY gene and GK regulation activity. This fundamental question remains unanswered, however, due to the lack of an accurate structural model for the complex of GK with ATP, glucose and Mg^2+^ (GK-Mg^2+^-ATP-glucose complex). In particular, an important residue in the binding pocket, Lys169, can undergo mutation (K169N) in MODY [20] and its functional role remains unknown.

Here, we report a study of the catalytic mechanism of GK by combining molecular modeling, molecular dynamics (MD) simulations, quantum mechanics/molecular mechanics (QM/MM) calculations, experimental mutagenesis and enzymatic kinetic analysis. The atomic structure of the glucose phosphorylation catalyzed by GK in aqueous solution was studied using protein substrate conformations obtained from the MD simulation. The simulation results are in agreement with the recent findings of mutagenesis experiments and related kinetic studies. MD simulations further revealed that Lys169 may play an essential role in both ligand binding and GK catalytic process. The computational prediction was verified by additional experimental mutagenesis and kinetic analysis. Based on these results, we propose an atomistic catalytic mechanism of GK for glucose phosphorylation using QM/MM calculations. Findings from this work provide a better understanding of the enzymatic mechanism of GK, and also a potential explanation of the pathogenic mechanism of MODY caused by the mutation in GK.

## Results

The main goal of this study is to investigate the catalytic process of human GK by using computational methods in conjunction with enzymatic assay. Such a study, however, needs an accurate structural model for the whole catalytic environment of GK, i.e. the three-dimensional (3D) structure of the complex of GK with ATP, glucose and Mg^2+^ (GK-Mg^2+^-ATP-glucose complex, designated as GMAG complex hereinafter). Accordingly, a 3D structural model of GMAG complex was constructed based on the crystal structures of GK and several other hexokinases by using molecular modeling. Following this, the dynamic conformation change of the catalytic site of GK was investigated by using MD simulations. Furthermore, the modeling and simulation results were validated using mutagenesis and enzymatic kinetic assays. Encouraged by the compatible results from theoretical prediction and experiments, we explored the GK catalytic mechanism for the glucose phosphorylation based on a stable conformation of GMAG complex derived from the MD simulations by using quantum mechanics/molecular mechanics (QM/MM) method.

### 3D Model of the GK-Mg^2+^-ATP-glucose (GMAG) Complex

The crystal structure of human GK had not been available until 2004 due to the high flexibility of the protein [21]. Moreover, it is easy for ATP to transfer its terminal phosphate to glucose *in situ*, making it difficult to gain accurate structural information of the catalytic environment of glucose inside GK, which plays a critical role in understanding the catalytic and regulation mechanisms of GK. Several models for GK catalytic environment have been published [22]–[27]. However, these models were built using homology modeling based on the X-ray crystal structure of human hexokinase type I [28], [29]. Although human hexokinase type I is homologous to GK with sequence identity of ∼55%, its three-dimensional (3D) structure is different, especially around the ATP binding pocket, as indicated by the structural superposition of the crystal structures of these two enzyme (Figure S1). The reliability of these GK catalytic models is therefore questionable. On the basis of the crystal structures of GK, now available, we built up a structural model for the GMAG complex (see *Materials and Method* section for detail). This model is more accurate than the previous models, and has also been validated by further MD and QM/MM calculations, as well as mutagenesis experiments and enzymatic assays (see discussion below).

**Figure 1:**
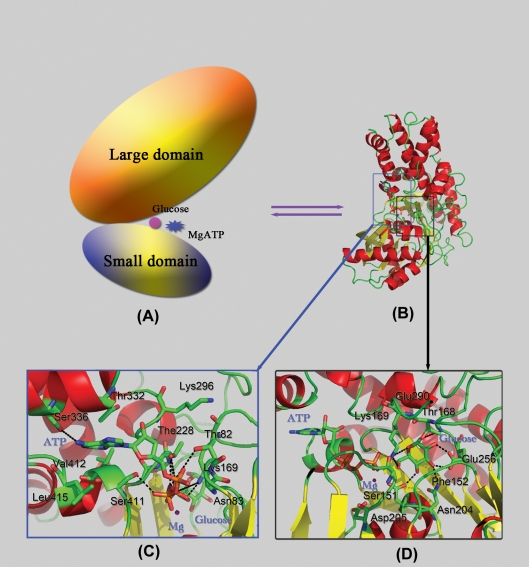
The structural model of GMAG complex. (A) 2D topological structure plot of GMAG. (B) 3D structure of GMAG. (C) Important interactions between ATP and GK. (D) Important interactions between glucose and GK. ATP, Mg^2+^, glucose and important residues in GK are displayed in stick and labeled. Black dashed lines represent hydrogen bonds or salt bridges.

Structurally, GK is composed of a large domain and a small domain; the channel-shaped active site for phosphorylation is located in the deep cleft between these two domains, as shown in Figures 1A and 1B. The Mg^2+^ ion and ATP (Mg^2+^-ATP) are predicted to bind to the left portion of the active site cleft, interacting with both domains [22]–[24]. The Mg^2+^-ATP binding site is formed by residues 78–83, 169, 225–228, 295–298, 331–336, 410–413, and 415–417 (Figure 1C). Meanwhile, the glucose resides in a small pocket composed of residues 80–81, 151–154, 168–169, 204–207, 225–232, 254–259, 287 and 290 (Figure 1D). Our 3D model of GMAG complex reveals that GK provides a favorable microenvironment for the phosphorylation of glucose. Several important hydrogen bonds between ligands (ATP, Mg^2+^ and glucose) and GK are observed in our model (Table S1), which are in agreement with a large number of mutagenesis analysis data [20], [30]–[34]. The γ-phosphate of ATP is close to the –O^6^H group of glucose with electrostatic interaction, indicating that the γ-phosphate is about to be transferred to glucose.

To study the stability of the reaction environment, a 10-ns molecular dynamics (MD) simulation was performed on the GMAG complex. The time evolutions of the weighted Root-Mean Square Deviations (wRMSD) for the atoms of GK, ATP and glucose from their initial positions (*t* = 0 ps) were monitored. The result indicates that all the reaction components are relatively stable except for the rotation of the –O^6^H group which causes an increase in wRMSD of glucose by 0.3 Å at 1 ns (Figure S2). The H-bond network amongst GK, ATP and glucose also reflects the stability of the phosphorylation environment. These H-bonds were mostly maintained during the 10-ns MD simulation as indicated by their occupancies (Table S1).

**Figure 2:**
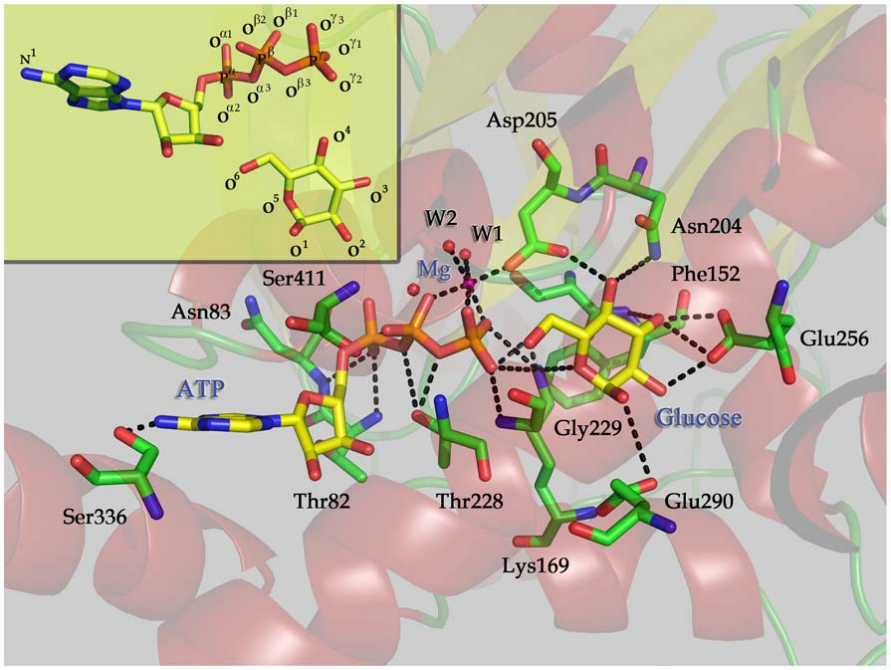
A close view of the configuration of glucose phosphorylation in the environment of GK active site. The structural model was constructed based on the snapshot at 2500 ps isolated from the 10-ns MD trajectory. W1 and W2 represent water molecules. ATP, Mg^2+^, glucose and important residues in GK are displayed in stick and labeled. Black dashed lines represent hydrogen bonds or salt bridges. Atomic numbering of ATP and glucose is displayed in the up-left panel.

Under the restriction of GK, ATP, Mg^2+^ and glucose form a highly advantageous configuration for the biochemical reaction (Figure 2). Mg^2+^ ion in the binding site octahedrally coordinates with the oxygen atoms of two water molecules, O^β1^, O^γ1^ and O^γ3^ oxygen atoms of ATP and the O^δ2^ atom of Asp205. Nine H-bonds are formed between ATP and residues of GK, i.e. the N^1^ atom of adenine with the –O^γ^H group of Ser336, the O^α2^ atom of ATP with the –NH groups of Thr82 and Asn83, the O^α1^ atom of ATP with the –O^γ^H group of Ser411, the O^α3^ and O^β3^ atoms of ATP with the –O^γ^H Thr228, the O^γ2^ atom of ATP with the –NH group of Gly229, and the O^γ1^ and O^γ2^ atoms of ATP with the –N^ζ^H^+^_3_ group of Lys169 (Figure 2 and Table S1). These H-bonds lead the ATP to adopt a conformation appropriate to coordinate with the Mg^2+^ ion and to interact with glucose. On the other side, glucose forms seven H-bonds with the GK residues, viz. the –O^4^H group of glucose with the N^δ1^ atom of Asn204 and the O^δ1^ atom of Asp205, the –O^3^H group of glucose with the O^ε1^ and O^ε2^ atoms of Glu256 and the –NH group of Phe152, the –O^1^H group of glucose with the O^ε1^ atom of Glu290, the O^5^ atom of glucose with the –N^ζ^H^+^_3_ group of Lys169 (Figure 2 and Table S1). Most importantly, the –O^6^H group of glucose directly hydrogen bonds to the O^γ2^ atom of ATP, producing the requisite complex for glucose phosphorylation. Residues detected in the naturally occurring mutations (e.g. K169N, T228M, E256K, S336L, and S411F) from MODY families [20], [30]–[32] are involved in the H-bond network for the binding of GK with glucose and ATP. Extensive work confirmed that S336L mutation decreased both the binding affinity of GK to ATP and the catalytic activity of the enzyme [33]. Several other site-directed mutants, including N204A, E256A and E290A, have been shown to alter GK's enzymatic kinetics by decreasing the binding affinity of glucose and lowering V_max_ in the catalytic process [31], [34]. These mutagenesis and enzymatic results demonstrate that our 3D model of GMAG complex is reasonable.

### Importance of Lys169 in the Phosphorylation of Glucose and Mutation Validation

Among the conserved residues hydrogen bonding to ATP and glucose, Lys169 is of special interest because its cationic end, –N^ζ^H^+^_3_, bridges ATP and glucose together through H-bonds, placing the γ-phosphorus (P^γ^) atom only 3.3 Å far way from the –O^6^H group of glucose (Figure 2). This implies that Lys169 might play an important role in the phosphorylation of glucose. Additionally, it was reported that Lys169Asn (K169N) is one of the naturally occurring mutations in the *GCK* gene associated with familial mild fasting hyperglycemia [20]. The K169N mutant was also shown to experience a partial loss of glucose binding [30].

To address the biological function of Lys169, we constructed a 3D structural model for the GK K169A mutant (GK_K169A_) in complex with ATP-Mg^2+^ and glucose, and two additional MD simulations of the ATP-Mg^2+^-GK_K169A_ and glucose-GK_K169A_ complexes were conducted. Binding free energies of ATP and glucose with wild-type GK and GK_K169A_ (Table S2) were then calculated using the MM-PBSA method encoded in AMBER (Version 8.0). Both ATP and glucose are able to bind tightly to the wild-type GK with calculated binding free energies of −18.67±4.59 and −44.06±3.94 kcal/mol, respectively. While neither ATP nor glucose can bind with GK_K169A_.

The MD simulations and binding free energy calculations indicate that Lys169 may dominate the binding of both ATP and glucose, and hence the K169A mutant possibly loses the catalytic function for the phosphorylation of glucose. To verify this conclusion, a bioassay on the K169A mutant was performed and result is shown in Table 1. The kinetic assay indicates that K169A is indeed an inactivating mutation; its enzymatic activity is completely abolished. Far-UV CD spectra and fluorescence emission spectra results demonstrated that the K169A mutant had well-defined secondary structures; thereby we can exclude the possibility that this mutation may have caused GK to undergo misfolding (Figures S3, S4, S5). Thus, the MD prediction, regarding the importance of Lys169 to the binding of ATP and glucose with GK and to the catalytic activity of GK as well was validated.

**Table 1:** Kinetic analysis of GK and GK_K169A_.

Mutants	Protein conc. (mg/ml)	V_max__Glucose (µM/min/mg)	S_Glucose 0.5 (mM)	Km_ATP (mM)	nH
LGK2-WT	2.24	3966.13±1107.69	11.64±0.83	0.30±0.01	1.40±0.12
K169A mutant	2.24	N/A	N/A	N/A	N/A

Data are means±SEM for wild-type hLGK2 and hLGK2 mutants. The results are means of kinetic analyses of three independent experiments of wild-type hLGK2 and hLGK2 mutants. nH is the Hill number.

### Lys169 Acts as a General Acid Catalyst

**Figure 3:**
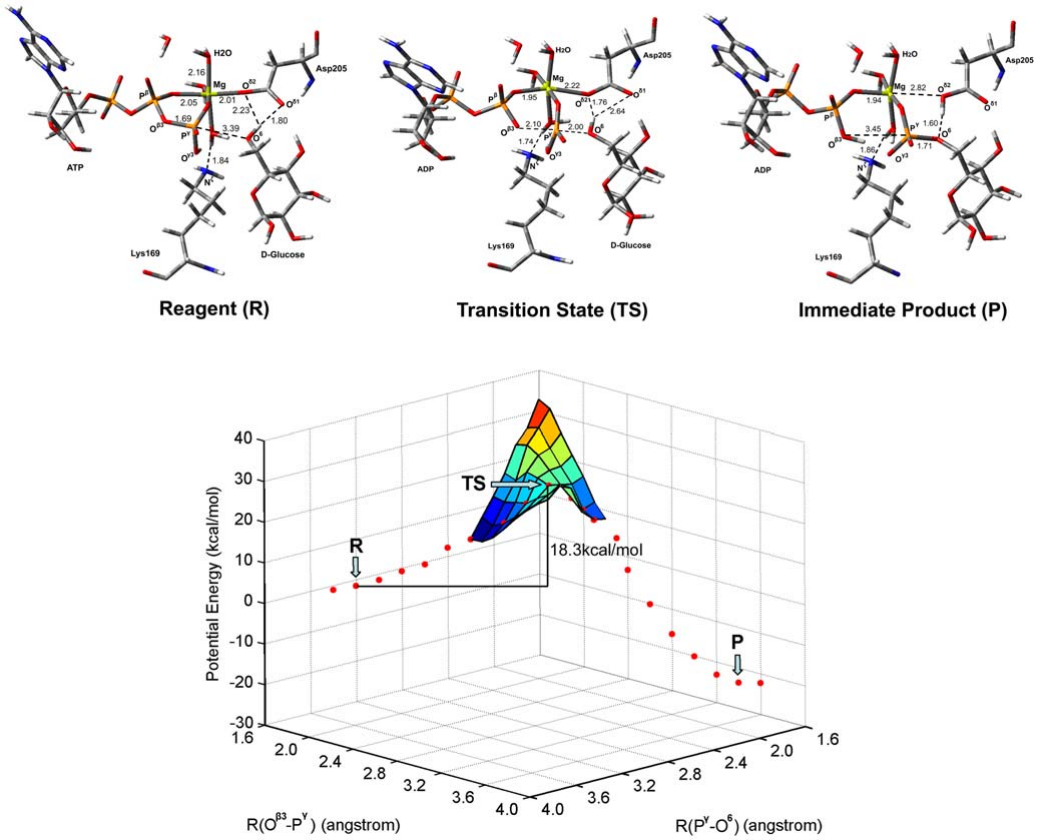
The potential energy surface of the reaction pathway corresponding to Scheme 1. The structures displayed at the top of the potential surface are the QM/MM optimized structures of the reagent (R), transition state (TS), and immediate product (P). For clarity, only the structures around the reaction center have been shown.

**Figure 4:**
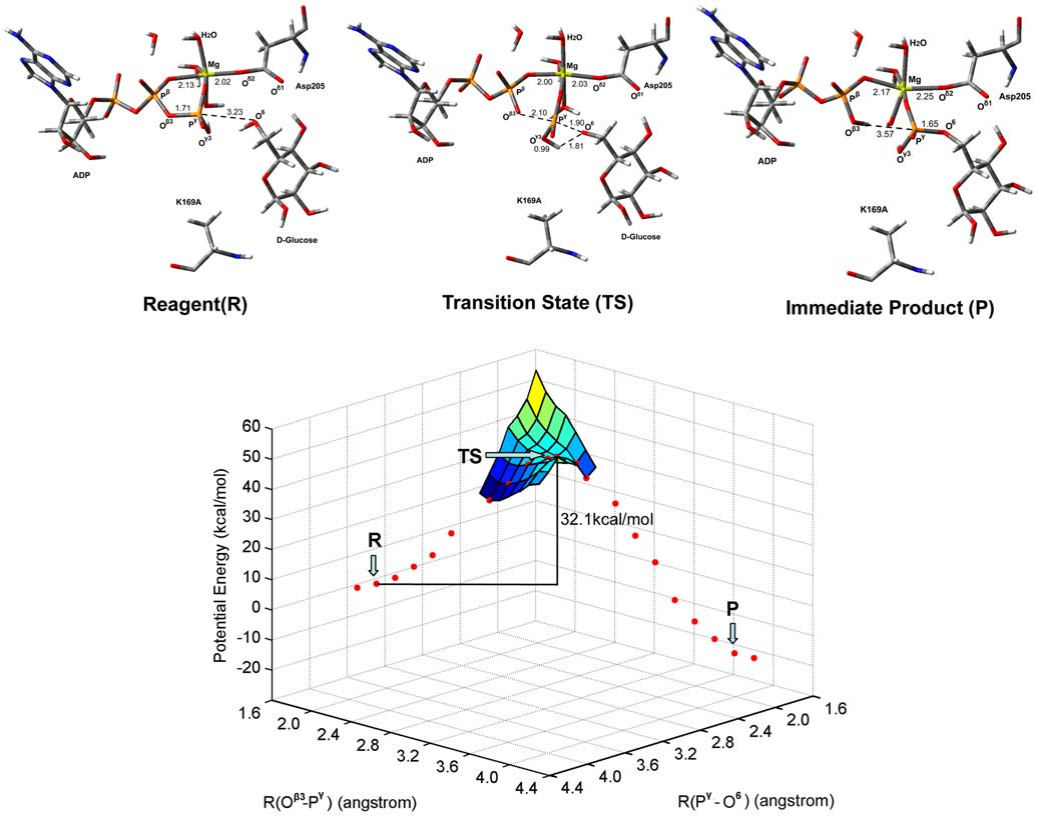
The potential energy surface of the reaction pathway corresponding to Scheme 2. The structures displayed at the top of the potential surface are the QM/MM optimized structures of the reagent (R), transition state (TS), and immediate product (P). For clarity, only the structures around the reaction center have been shown.

To further figure out the role of Lys169 in enzymatic catalysis, the reaction mechanism of glucose phosphorylation inside the active site of GK was investigated by using the QM/MM approach. The system for QM/MM simulation was constructed based on the snapshot at 2500 ps of the MD trajectory. The QM region was composed of Mg^2+^, water molecules coordinating with Mg^2+^, important groups of ATP, glucose, Lys169 and Asp205; the remainder of GK was included in the MM region (Figure S6). The partitioning scheme for QM and MM regions is described in the *Materials and Methods* section and Figure S6. We designate this structure as reagent system. For the reagent system, the initial coordination configuration of Mg^2+^ with waters, ATP and glucose and the hydrogen bonding pattern amongst ATP, glucose, Lys169 and Asp205 have been described above (Figure 2). ONIOM, a QM/MM method encoded in Gaussion03 [35], was used for all the QM/MM calculations (Figure 3 and 4).

**Figure 5:**
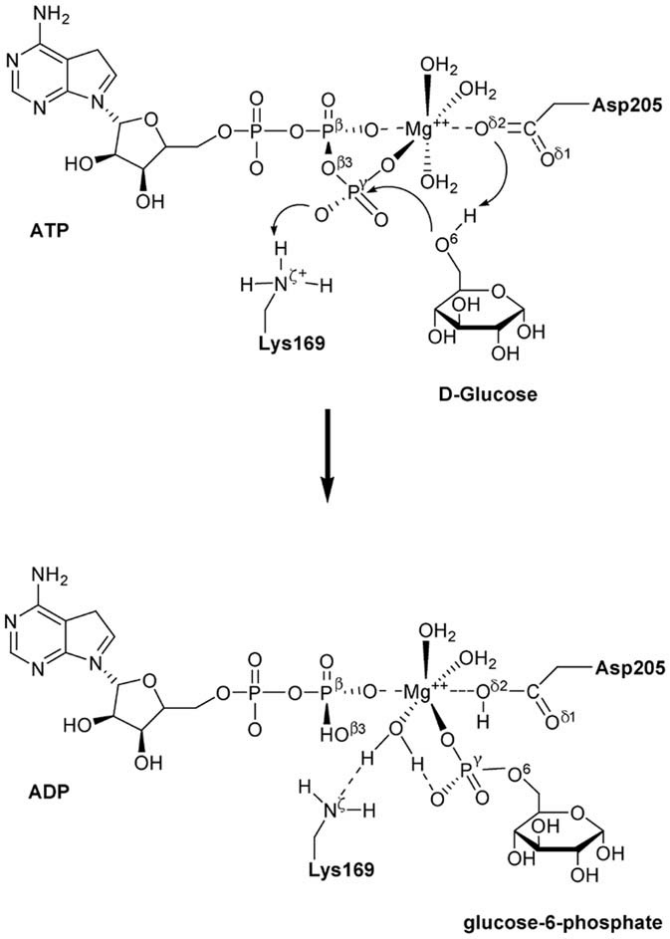
Reaction mechanism of glucose phosphorylation catalyzed by the wild-type GK.

The QM/MM optimized geometry of the reagent system showed that Mg^2+^ octahedrally coordinates with the O^β1^and O^γ1^ atoms of ATP, the O^δ2^ atom of Asp205 and three oxygen atoms from three water molecules (Figure 3). The major difference of the optimized structure for the reaction center from the initial structure obtained by MD simulation is that one water molecule substituted the O^γ3^ atom to coordinate with the Mg^2+^ ion. QM optimization resulted in important structural changes viz. one proton of the –N^ζ^H^+^_3_ group of Lys169 transferred to the O^γ3^ atom, and the resulting –N^ζ^H_2_ group formed a H-bond with the new water molecule coordinating with the Mg^2+^ ion; on the other hand, the –O^6^H group of glucose adjusted its direction to point toward the O^δ1^ atom of Asp205, forming two strong H-bonds with the O^δ1^ and O^δ2^ atoms of Asp205. This structural reorganization made the –O^6^H group more propitious to attack the P^γ^ atom of ATP, implying that Lys169 might act as an acid catalyst and Asp205 as a base catalyst in the phosphorylation of glucose. Thus, we propose a mechanism for the glucose phosphorylation catalyzed by GK as Scheme 1 (Figure 5). Along this reaction path, the energies of the reagent (R), transition state (TS), and immediate product (P) were determined by two-dimensional QM/MM potential energy surface by defining the distances of *R*(O^β3^―P^γ^) and *R*(P^γ^―O^6^) as the reaction coordinates (Figure 3). In the optimized reagent, *R*(O^β3^―P^γ^) = 1.69 Å and *R*(P^γ^―O^6^) = 3.39 Å; and in the optimized immediate product, the O^β3^―P^γ^ bond is broken and the P^γ^―O^6^ is formed, *R*(P^γ^―O^6^) = 1.71 Å. The calculated potential energy barrier of Scheme 1 is Δ*E* ^≠^ = 18.3 kcal/mol.

The structure of the transition state (TS) in Scheme 1 was determined by adiabatic mapping at the QM/MM level (Figure 3). In the TS, *R*(O^β3^―P^γ^) = 2.1 Å and *R*(P^γ^―O^6^) = 2.0 Å, and the γ-phosphate (–P^γ^O_3_) is turned to be in a plane. This structure clearly illustrates that the P^γ^―O^6^ bond is partially formed and the O^β3^―P^γ^ bond is partially broken. The overall reaction is calculated to be exothermic by Δ*E* = −22.1 kcal/mol.

**Figure 6:**
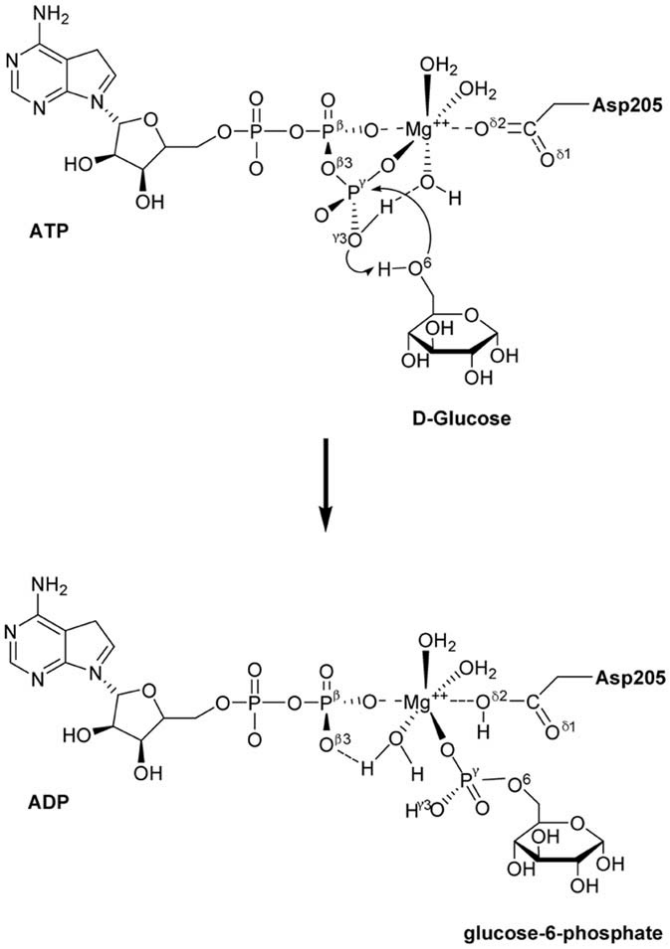
Reaction mechanism of glucose phosphorylation catalyzed by the GK_K169A_ mutant.

To further address the catalytic role of Lys169, we investigated the reaction mechanism of glucose phosphorylation catalyzed by the GK_K169A_ mutant employing the QM/MM method. Compared with the optimized structure of GK reagent system, the major difference for the ATP-GK_K169A_-glucose reaction center is that the –O^6^H group of glucose doesn't form H-bond with either O^δ1^ or O^δ2^ atom of Asp205, but forms a strong H-bond with the O^γ3^ atom of ATP. This optimized structure implies an alternative path for the glucose phosphorylation as Scheme 2 (Figure 6): the proton of the –O^6^H group transfers to the O^γ3^ atom of ATP while the O^6^ atom attacks the P^γ^ atom of ATP, and at the same time the O^β3^―P^γ^ bond is broken. According to this reaction path, we obtained the structures and energies of reagent (R), transition state (TS), and immediate product (P) by calculating the two-dimensional QM/MM potential energy surface; the distances of *R*(O^β3^―P^γ^) and *R*(P^γ^―O^6^) were defined as the reaction coordinates. The result is shown in Figure 4. For the TS, *R*(O^β3^―P^γ^) = 2.1 Å and *R*(P^γ^―O^6^) = 1.9 Å. Remarkably, the calculated potential energy barrier of this reaction path is Δ*E* ^≠^ = 32.1 kcal/mol, which is ∼14 kcal/mol higher than that of the reaction catalyzed by the wild-type GK. This result indicates that the phosphorylation of glucose could not occur without the assistance of Lys169.

## Discussion

As mentioned above, GK is a key enzyme that phosphorylates glucose and triggers glucose utilization and metabolism. As an attractive drug target for discovering anti-diabetes drug, the importance of this enzyme has been appreciated. However, the unique catalytic mechanism of GK still remains unclear [17], [18]. Several mutants of GK with the MODY phenotype have been identified recently; in particular, it was found that K169N is a naturally occurring mutation (K169N) in MODY [20]. In this study, we have investigated the phosphorylation mechanism of glucose catalyzed by GK, and revealed that Lys169 is a crucial residue for glucose phosphorylation.

By using molecular modeling and simulation methods, we constructed a 3D structural model for the complex of GK with ATP, glucose and Mg^2+^ (GMAG complex). This structural model is more reliable than previously published models [22]–[27], [36] because it was constructed based on the crystal structures of human GK. In particular, this model provides a more realistic reaction environment for the phosphorylation of glucose. The validity of the structural model of GMAG was confirmed by the agreement between the available experimental mutagenesis and enzymatic data for GK and the important interactions of GK with ATP, glucose and Mg^2+^ identified through computation [20], [30]–[34].

Both ATP and glucose interact with the residues lining the binding pocket of GK through H-bonds (Figure 2). Among these residues, Lys169 plays a significant role, because it forms H-bonds with both ATP and glucose. This indicates that Lys169 contributes to the binding of GK with either ATP or glucose or both. Computational simulations demonstrated that both ATP and glucose are able to bind to the wild-type GK, and neither ATP nor glucose can bind to the GK_K169A_ mutant (Table S2). Indeed, the experimental mutagenesis and enzymatic kinetic assay validated the computational prediction, i.e. the mutant of K169A could not bind to ATP and glucose and its enzymatic activity is completely lost (Table 1).

The QM/MM calculations on the mechanisms of glucose phosphorylation catalyzed by both GK and GK_K169A_ mutant further revealed the role of Lys169 in catalysis. During the phosphorylation process of glucose, in addition to enhancing the binding of ATP and glucose with GK, Lys169 also acts as a general acid catalyst, providing a proton to protonate the O^γ3^ atom of ATP; at the same time Asp205 acts as a general base catalyst, extracting the proton from the –O^6^H group of glucose (Scheme 1). On the other hand, ATP and glucose bind to the GK_K169A_ mutant with a different model than with the wild-type GK (Figures 3 and 4), whereby the phosphorylation of glucose adopts a different mechanism (Scheme 2). The QM/MM calculated activation energy of Scheme 2 is ∼14 kcal/mol higher than that of Scheme 1. This suggests that the GK_K169A_ mutant has significantly reduced catalytic activity than its wild-type form. The calculated result is in good agreement with our mutagenesis data (Table 1). Moreover, the QM/MM result also indicates that Asp205 of GK loses its function as a general base catalyst in the glucose phosphorylation when Lys169 is absent (Figure 4).

The agreement between computation and experimental results validates the essential role of Lys169 in the phosphorylation of glucose. In summary, Lys169 plays at least three functional roles in the metabolism of glucose: (1) it enhances the binding of GK to both ATP and glucose; (2) it bridges ATP and glucose together; and (3) it acts as a general acid catalyst and directly participates in glucose phosphorylation. These results are important for understanding the catalytic mechanism of GK and the cause of the pathogenic mechanism of MODY due to the GK mutation[17], [18]. Obviously, asparagine cannot act as a general acid catalyst because its side chain is incapable of providing a proton to ATP, as shown in Figure S7. Our study thus provides an explanation of why the catalytic activity of the mutant GK (K169N) in the MODY is lower than that of the wild-type GK.

## Materials and Methods

### Structural Model

On the basis of crystal structures GK (the active human GK with glucose, PDB entry 1V4S) and two hexokinases (human brain hexokinase complexed with glucose and phosphate, PDB entry 1HKC, and monomeric hexokinase I complexed with ADP, PDB entry 1DGK) as templates, the whole structural model of GMAG complex (GK-Mg^2+^-ATP-glucose complex) was constructed by using a heuristic approach. The crystal structure of GK-glucose complex (1V4S) was used as a basal structure. The ATP conformation was generated according to the structure of ADP in the crystal structure of 1DGK, and the coordination configuration of Mg^2+^ with ATP (Mg^2+^-ATP) was constructed based on the coordination state of Mg^2+^ in the crystal structure of 1HKC. Then the Mg^2+^-ATP was “grafted” to the binding pocket of GK, and the primary position of Mg^2+^-ATP was decided through structural superimposition for the crystal structures of the three enzymes using the Homology module of Insight2005 (Accelrys, San Diego, CA). For the initial structure of the GMAG complex, residues within a radius of 6.5 Å around the binding site were optimized by energy minimization using the AMBER force field implemented in the Sybyl6.8 (Tripos, St. Louis, MO) with following parameters: a distance-dependent dielectric function, non-bonded cutoff of 8 Å, Amber charges for the protein, Gastieger-Hückel charges for ATP and glucose. The structure was minimized by simplex method first, followed by Powell method to an energy gradient<0.05 kcal/(mol·Å). The structural model of G_K169A_MAG and G_K169N_MAG complex were simply constructed by replacing Lys169 with Ala and Asn respectively, followed by minimization using the same protocol as in the structural optimization for the model of GMAG.

### Molecular Dynamics Simulation and Binding Free Energy Calculation

MD simulations were performed on the GMAG and G_K169A_MAG complexes. Before simulations, each complex was put into a suitably sized box, of which the minimal distance from the protein to the box wall was 15 Å. Then the box was solvated with the SPC (Simple Point Charge) water model [37]. Each complex/water system was submitted to energy minimization. Afterwards, counterions were added to the system to provide a neutral simulation system. The whole system was subsequently minimized again. The charges of the atoms of ATP and glucose were calculated by using the RESP method [38] encoded in the AMBER suite of programs [39] at the level of RHF/6-31G*. Covalent and non-bonded parameters for the ligand atoms were assigned, by analogy or through interpolation, from those already present in the AMBER force field [40].

MD simulations were carried out using the AMBER package (Version 8.0) with constant temperature, constant pressure (NPT), and periodic boundary conditions. The AMBER Parm99 force field [40] was applied for the proteins. The Particle Mesh Ewald method [41] was used to calculate the long-range electrostatics interactions. The non-bonded cutoff was set to 12.0 Å, and the non-bonded pairs were updated every 25 steps. The SHAKE method [42] was applied to constrain all covalent bonds involving hydrogen atoms. Each simulation was coupled to a 300 K thermal bath at 1.0 atm of pressure (1 atm = 101.3 kPa) by applying the algorithm of Berendsen *et al.* [43]. The temperature and pressure coupling parameters were set as 1 ps. An integration step of 2 fs was set up for the MD simulations.

On the basis of the equilibrated dynamic trajectory, the binding free energy of each system (GMAG, GK_K169A_-Glucose and GK_K169A_-Mg^2+^-ATP) was calculated by using the MM-PBSA method encoded in the AMBER 8.0 program. Coordinates from the trajectory were used every 20 ps, and the MM-PBSA calculation was performed on each of them using the AMBER 8.0 program. For each snapshot collected during the simulations, binding free energy (Δ*G*_binding_) was calculated using Eq. (1):
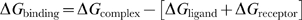
(1)where Δ*G*_complex_, Δ*G*_ligand_ and Δ*G*_receptor_ are the free energies of the complex and two components of interaction. Each free energy term in Eq. (1) in AMBER 8.0 was calculated with the absolute free energy in gas phase (*E*_gas_), the solvation free energy (Δ*G*_solvation_) and the entropy term (TΔ*S*) using Eq. (2):
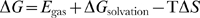
(2)
*E*_gas_ is the sum of the internal strain energy (*E*_int_), van der Waals energy (*E*_vdw_) and electrostatic energy (*E*_electrostatic_) (Eq. (3)). *E*_int_ is the energy associated with vibrations of covalent bonds and bond angles, rotation of single bond torsional angles (Eq. (4)).
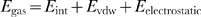
(3)

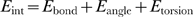
(4)


The solvation free energy, Δ*G_s_*_olvation_, is approximated as the sum of the polar contribution (*G*_PB_) and nonpolar contribution (*G*_nonpolar_) using a continuum representation of the solvent:
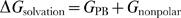
(5)


The polar contribution (*G*_PB_) to the solvation energy was calculated using PB model in sander module. The nonpolar contributions (*G*_nonpolar_) were estimated using a simple equation: *G*_nonpolar_ = γ×SASA+*b* (kcal/mol). SASA is the solvent-accessible surface area estimated using the MSMS algorithm with probe radius of 1.4 Å. The surface tension proportionality constant γ and the free energy of nonpolar solvation for a point solute *b* were set to 0.00542 kcal/(mol Å^2^) and 0.092 kcal/mol, respectively.

The entropy calculation is extremely time-consuming for large systems. In addition, the main aim of calculating the binding free energy was to address the influence of mutations to the binding affinity. Site-directed mutation may not result in dramatic conformational changes for the protein and it was assumed that entropy contributes little to the relative binding free energy for the binding of mutant in comparison with the wild-type protein. Therefore, in this study, Δ*G*_binding_ (without term of -TΔS) was estimated to address the mutation effect to of the binding free energy.

### QM/MM Calculation

QM/MM calculations were performed by using a two-layered ONIOM method encoded in the Gaussian03 program [35]. The ONIOM method is a hybrid computational method developed by Morokuma and coworkers that allows different levels of theory to be applied to different parts of a molecular system [44]–[52]. In the two-layered ONIOM method, the molecular system under study is divided into an inner layer and an outer layer. The inner layer consists of the most critical elements of the system, and the rest of the system comprises the outer layer. In the terminology of Morokuma and co-workers, the full system is called “real” and is treated with a low level of theory. The inner layer is termed “model” and is treated with both the low level of theory and a high level of theory. The total ONIOM energy *E*_ONIOM_ is given as following:
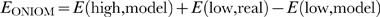
(6)where *E*(high, model) is the energy of the inner layer (plus the link atoms) at the high level of theory, *E*(low, real) is the energy of the entire system at the low level of 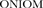 theory, and *E*(low, model) is the energy of the model system at the low level of theory. Thus, the ONIOM method allows one to perform a high-level calculation on just a small, critical part of the molecular system and incorporate the effects of the surrounding elements at a lower level of theory to yield a consistent energy expression on the full system.

The minimized structure optimized using the AMBER Parm99 force field was further optimized at the ONIOM (B3LYP/6-31G*: Amber) level. The quantum mechanics (QM) region includes the β, γ-phosphate group of fully unprotonated ATP, four water molecules including two coordinated water molecules, Mg^2+^ ion, the hydroxymethyl group (–CH_2_–O^6^H) of glucose, the methyleneammonium group (–CH_2_–N^ζ^H^+^_3_) of Lys169 and the anionic carboxymethyl group (–CH_2_–COO^−^) of Asp205 for a total of 40 atoms (Figure S6). The QM region was calculated by using the density functional theory with the B3LYP exchange-correlation functional and 6–31G* basis set. The remainder of the system (MM region) was treated by using the AMBER Parm99 force field. A total of 7,147 atoms were included for the QM/MM calculations by using the ONIOM module as implemented in Gaussian 03. The electrostatic interactions between the QM and MM regions were calculated by using the electronic embedding method, which treats the polarization of the QM region by the MM region with scaled partial atomic charges of MM atoms, and the response of QM region with Merz-Singh-Kollman scheme for charge fitting so as to produce the changing partial charges of the QM atoms. The charge for the QM region was −1, and the charge for the MM was 1, therefore the total system remained neutral.

### Expression, Purification and Kinetic Analysis on GK and Its K169A Mutant

*Escherichia coli* (*E. coli*) host strain M15 was purchased from Qiagen (Valencia, CA). All chemicals were of reagent grade or ultra-pure quality, and commercially available. Human liver glucokinase isoform 2 (hLGK2) DNA sequence was prepared by total gene synthesis method (Sangon, Shanghai, China).

Human liver GK isoform 2 (hLGK2) plasmid was digested with restriction endonucleases BamHI and SalI (NEB), and cloned into a prokaryotic expression vector pQE-30 (Qiagen) to produce the recombinant plasmid pQE-30-hLGK2, containing a N-terminal six-histidine tag to facilitate purification. The recombinant clone pQE-30-hLGK2 was confirmed by sequencing. The pQE-30-hLGK2 mutant K169A was constructed by PCR (polymerase chain reaction)-based site-directed mutagenesis using pQE-30-hLGK2 as template. To make the single-amino-acid-change mutants, the following primer (F: forward; R: reverse) was used during PCR:

K169A: F: 5′ CCTTCTCAACTGGACC**gcg**GGCTTCAAGGCC3′

R: 5′ GGCCTTGAAGCC**cgc**GGTCCAGTTGAGAAGG3′

Detailed procedures referred to QuikChange® Site-Directed Mutagenesis Kit (Stratagene) instruction manual. The mutant clones pQE-30-hLGK2 K169A was also confirmed by sequencing.

Expression and purification of the recombinant protein was performed according to His-Bind Kits (Novagen, San Diego, CA). The recombinant clones pQE-30-hLGK2 and pQE-30-hLGK2 K169A were separately transformed into *E. coli* strain M15 that grew in LB media supplemented with 100 µg/mL ampicillin and 50 µg/mL kanamycin at 37 °C. Expressions of pQE-30-hLGK2 was induced by 0.3 mM IPTG (Isopropyl-β-D-thiogalactopyranoside) when the OD_600_ reached 0.6 at 25 °C for an additional 16–20 h. The hLGK2 cells were harvested by centrifugation and suspended in Buffer A (20 mM Tris-HCl, pH 8.0, 500 mM NaCl, 10 mM imidazole), stored at −80 °C after centrifugation until use. Packed cells were suspended in Buffer A plus 1 mM PMSF (Phenylmethanesulfonylfluoride), after sonication treatment on ice, the mixture was centrifuged to yield a clear supernatant, which was loaded onto a column with Ni-NTA resin (Qiagen) pre-equilibrated in Buffer A (20 mM Tris-HCl, pH 8.0, 500 mM NaCl, 10 mM imidazole). The column was washed with 8–10 column volumes of Buffer B (20 mM Tris-HCl, pH 8.0, 500 mM NaCl, 20 mM imidazole) and eluted with Buffer C (20 mM Tris-HCl, pH 8.0, 500 mM NaCl, 150 mM imidazole), then the soluble hLGK2 fractions were pooled separately and dialyzed against Buffer D (25 mM HEPES, pH 7.1, 25 mM KCl, 2 mM MgCl_2_, 1 mM DTT) to remove imidazole. The hLGK2 protein was thus concentrated by ultrafiltration with Amicon centrifugal filter device to appropriate concentration. All procedures including purification, dialysis and concentration were performed at 4 °C. Protein concentration was determined by Bradford assay using bovine serum albumin (BSA) as standard. The expressions, purifications and the subsequent procedures of hLGK2 mutant K169A protein were the same as that of hLGK2 protein.

The recombinant proteins of hLGK2 and hLGK2 mutant K169A was subjected to kinetic analysis (V_max_, K_m-ATP_, S_0.5_ and nH) according to the published procedure reported by Grimsby *et al*. [53]. The assay solution contained 25 mM HEPES (pH 7.1), 25 mM KCl, 2 mM MgCl_2_, 1 mM ATP (0.01 mM, 0.03 mM, 0.06 mM, 0.10 mM, 0.20 mM, 0.30 mM, 0.40 mM, 0.50 mM, 0.60 mM, 0.80 mM, 1.0 mM and 2.0 mM in K_m-ATP_ assay), 1 mM DTT, 1 mM NAD, 0.1% BSA, 5 U/ml glucose-6-phosphate dehydrogenase from *Leuconostoc mesenteroides*, 5 mM glucose (0.1 mM, 0.4 mM, 1.0 mM, 2.0 mM, 5.0 mM, 10.0 mM, 20.0 mM, 64.0 mM, 100.0 mM, 200 mM, 300 mM and 400 mM in S_0.5_ assay) and 18.7 ug/ml hLGK2 or hLGK2 mutants fusion protein in a total volume of 0.12 ml. All assays at 25 °C were conducted in a 96-well plate system (Benchmark™ Plus Microplate Spectrophotometer) by measuring the increase in absorbance at 340 nm.

### CD spectra of hLGK2 WT and hLGK2 mutant proteins

CD spectra of hLGK2 WT and K169A mutant proteins were recorded on a Jasco-810 spectropolarimeter equipped with a thermal controller. The protein sample was prepared in 20 mM NaH_2_PO_4_, pH 7.1, at 25°C with protein concentration of 5 µM. Far-UV CD spectra from 190 to 250 nm were collected with 1 nm bandwidth using a 0.1 cm path length cuvette and normalized by subtracting the base line recorded for the buffer under identical conditions. Each measurement was repeated three times, and the final result was the average of the three independent scans.

### Fluorescence Spectra of Wild-type hLGK2 and hLGK2 Mutant Proteins

Fluorescence experiments of hLGK2 WT and mutant hLGK2 proteins K169A were carried out on a Hitachi F-2500 fluorescence spectrophotometer. The samples were prepared in 20 mM NaH_2_PO_4_ solution at pH 7.1 with protein concentration at 5 µM. The fluorescence emission spectra from 300 to 400 were collected after excitation at 280 nm. Fluorescence emission spectra of proteins were determined using a 1 cm path quartz cuvette at 25°C. The spectral band width was 5 nm for excitation and 10 nm for emission. The maximal emission wavelength was calculated by the affiliated software of the F-2500 spectrophotometer.

## Supporting Information

Table S1 Hydrogen bonds existing in the GMAG complex model and their occupancies in the 10-ns MD simulation.
(0.05 MB DOC)

Table S2 Predicted binding free energies of GK and GKK169A mutant with glucose and ATP.
(0.03 MB DOC)

Figure S1 Comparison between GK and homology hexokinases I. (A) The sequence alignment of GK and hexokinases I. (B) Local conformation of residues around ATP binding pocket in the superimposed crystal structures 1DGK(hexokinases I), 1HKC (hexokinases I) and 1V4S(GK). The ATP is colored by cyan. The carbons in 1DGK, 1HKC and 1V4S are colored by yellow, pink and green, respectively.
(4.35 MB TIF)

Figure S2 Time dependencies of the weighted Root-Mean-Square Deviations (wRMSDs) for the atoms of glucose (A), ATP (B) and GK (C) from their initial positions during the 10-ns MD simulation. (D) Residue fluctuations obtained by averaging atomic fluctuations over the MD simulation (black curve) and by computing the value from experimentally derived B factors (red curve) for GK crystal structure. (E) Time dependencies of energy during the 10-ns MD simulation.
(1.89 MB TIF)

Figure S3 SDS-PAGE for the purified wild-type hLGK2 and its K169A mutant proteins (M: Marker; WT: wild-type hLGK2; K169A: hLGK2 K169A mutant). The molecular weight of wild-type hLGK2 and hLGK2 K169 mutant proteins were evaluated as about 52 kDa indicating by the standard protein markers, which is in agreement with the calculated mass (52 kDa).
(8.41 MB TIF)

Figure S4 CD spectra of recombinant wild-type hLGK2 and K169A mutant proteins. Far-UV CD spectra of recombinant proteins were monitored at the concentration of 5 µM/L in 20 mM NaH_2_PO_4_ buffer (pH 7.1).
(0.66 MB TIF)

Figure S5 Fluorescence emission spectra of recombinant hLGK2 (wild-type) and K169A mutant proteins. Fluorescence emission spectra of the recombinant proteins were monitored at the concentration of 5 µM/L in 20 mM NaH_2_PO_4_ buffer (pH 7.1).
(4.27 MB TIF)

Figure S6 The partitioning scheme of QM and MM regions for the GMAG complex in the QM/MM calculations. Atoms in the QM region are displayed in ball and stick. ATP, Mg^2+^, glucose and important residues in GK are labeled.
(4.45 MB TIF)

Figure S7 A close view of the configuration of K169N in the environment of GK active site.
(0.62 MB TIF)

## References

[1] Al-Hasani H, Tschop MH, Cushman SW (2003). Two birds with one stone: novel glucokinase activator stimulates glucose-induced pancreatic insulin secretion and augments hepatic glucose metabolism.. Mol Interv.

[2] Van Schaftingen E (1994). Short-term regulation of glucokinase.. Diabetologia.

[3] Matschinsky FM, Glaser B, Magnuson MA (1998). Pancreatic beta-cell glucokinase: closing the gap between theoretical concepts and experimental realities.. Diabetes.

[4] Vionnet N, Stoffel M, Takeda J, Yasuda K, Bell GI (1992). Nonsense mutation in the glucokinase gene causes early-onset non-insulin-dependent diabetes mellitus.. Nature.

[5] Froguel P, Zouali H, Vionnet N, Velho G, Vaxillaire M (1993). Familial hyperglycemia due to mutations in glucokinase. Definition of a subtype of diabetes mellitus.. N Engl J Med.

[6] Glaser B, Kesavan P, Heyman M, Davis E, Cuesta A (1998). Familial hyperinsulinism caused by an activating glucokinase mutation.. N Engl J Med.

[7] Christesen HB, Jacobsen BB, Odili S, Buettger C, Cuesta-Munoz A (2002). The second activating glucokinase mutation (A456V): implications for glucose homeostasis and diabetes therapy.. Diabetes.

[8] Grossbard L, Schimke RT (1966). Multiple hexokinases of rat tissues. Purification and comparison of soluble forms.. J Biol Chem.

[9] Gloyn AL, Odili S, Zelent D, Buettger C, Castleden HA (2005). Insights into the structure and regulation of glucokinase from a novel mutation (V62M), which causes maturity-onset diabetes of the young.. J Biol Chem.

[10] Kantrowitz ER, Lipscomb WN (1988). Escherichia coli aspartate transcarbamylase: the relation between structure and function.. Science.

[11] Barford D, Johnson LN (1989). The allosteric transition of glycogen phosphorylase.. Nature.

[12] Schirmer T, Evans PR (1990). Structural basis of the allosteric behaviour of phosphofructokinase.. Nature.

[13] Iwata S, Kamata K, Yoshida S, Minowa T, Ohta T (1994). T and R states in the crystals of bacterial L-lactate dehydrogenase reveal the mechanism for allosteric control.. Nat Struct Biol.

[14] MacRae IJ, Segel IH, Fisher AJ (2002). Allosteric inhibition via R-state destabilization in ATP sulfurylase from Penicillium chrysogenum.. Nat Struct Biol.

[15] Kamata K, Mitsuya M, Nishimura T, Eiki J, Nagata Y (2004). Structural basis for allosteric regulation of the monomeric allosteric enzyme human glucokinase.. Structure.

[16] Zhang J, Li C, Chen K, Zhu W, Shen X (2007). Conformational transition pathway in the allosteric process of human glucokinase.. Proc Natl Acad Sci USA.

[17] Zelent B, Odili S, Buettger C, Shiota C, Grimsby J (2008). Sugar binding to recombinant wild-type and mutant glucokinase monitored by kinetic measurement and tryptophan fluorescence.. Biochem J.

[18] Printz RL, Granner DK (2005). Tweaking the glucose sensor: adjusting glucokinase activity with activator compounds.. Endocrinology.

[19] Postic C, Shiota M, Magnuson MA (2001). Cell-specific roles of glucokinase in glucose homeostasis.. Recent Prog Horm Res.

[20] Gloyn AL (2003). Glucokinase (GCK) mutations in hyper- and hypoglycemia: maturity-onset diabetes of the young, permanent neonatal diabetes, and hyperinsulinemia of infancy.. Hum Mutat.

[21] Kamata K, Mitsuya M, Nishimura T, Eiki J, Nagata Y (2004). Structural basis for allosteric regulation of the monomeric allosteric enzyme human glucokinase.. Structure.

[22] Mahalingam B, Cuesta-Munoz A, Davis EA, Matschinsky FM, Harrison RW (1999). Structural model of human glucokinase in complex with glucose and ATP: implications for the mutants that cause hypo- and hyperglycemia.. Diabetes.

[23] Marotta DE, Anand GR, Anderson TA, Miller SP, Okar DA (2005). Identification and characterization of the ATP-binding site in human pancreatic glucokinase.. Arch Biochem Biophys.

[24] Miller SP, Anand GR, Karschnia EJ, Bell GI, LaPorte DC (1999). Characterization of glucokinase mutations associated with maturity-onset diabetes of the young type 2 (MODY-2): different glucokinase defects lead to a common phenotype.. Diabetes.

[25] St Charles R, Harrison RW, Bell GI, Pilkis SJ, Weber IT (1994). Molecular model of human beta-cell glucokinase built by analogy to the crystal structure of yeast hexokinase B.. Diabetes.

[26] Xu LZ, Weber IT, Harrison RW, Gidh-Jain M, Pilkis SJ (1995). Sugar Specificity of Human .beta.-Cell Glucokinase: Correlation of Molecular Models with Kinetic Measurements.. Biochemistry.

[27] Bork P, Sander C, Valencia A (1992). An ATPase domain common to prokaryotic cell cycle proteins, sugar kinases, actin, and hsp70 heat shock proteins.. Proc Natl Acad Sci USA.

[28] Aleshin AE, Kirby C, Liu X, Bourenkov GP, Bartunik HD (2000). Crystal structures of mutant monomeric hexokinase I reveal multiple ADP binding sites and conformational changes relevant to allosteric regulation.. J Mol Biol.

[29] Aleshin AE, Zeng C, Bartunik HD, Fromm HJ, Honzatko RB (1998). Regulation of hexokinase I: crystal structure of recombinant human brain hexokinase complexed with glucose and phosphate.. J Mol Biol.

[30] Janne M, Lise B, Oddmund S, Pål RN, Torgeir F (2008). Catalytic activation of human glucokinase by substrate binding-residue contacts involved in the binding of D-glucose to the super-open form and conformational transitions.. FEBS J.

[31] Pilkis SK, Weber IT, Harrison RW, Bell GI (1994). Glucokinase: structural analysis of a protein involved in susceptibility to diabetes.. J Biol Chem.

[32] Barrio R (2002). Nine Novel Mutations in Maturity-Onset Diabetes of the Young (MODY) Candidate Genes in 22 Spanish Families.. J Clin Endocrinol Metab.

[33] Davis EA, Cuesta-Muñoz A, Raoul M, Buettger C, Sweet I (1999). Mutants of glucokinase cause hypoglycaemia- and hyperglycaemia syndromes and their analysis illuminates fundamental quantitative concepts of glucose homeostasis.. Diabetologia.

[34] Sener A, Malaisse-Lagae F, Xu LZ, Pilkis SJ, Malaisse WJ (1996). Anomeric specificity of the native and mutant forms of human β-Cell glucokinase.. Arch Biochem Biophys.

[35] Frisch MJ (2003).

[36] Gidh-Jain M, Takeda J, Xu LZ, Lange AJ, Vionnet N (1993). Glucokinase mutations associated with non-insulin-dependent (type 2) diabetes mellitus have decreased enzymatic activity: implications for structure/function relationships.. Proc Natl Acad Sci USA.

[37] Berendsen HJC, Postma JPM, van Gunsteren WF, Hermans J (1981). Intermolecular Forces..

[38] Bayly CI, Cieplak P, Cornell WD, Kollman PA (1993). A well-behaved electrostatic potential based method using charge restraints for deriving atomic charges: the RESP model.. J Phys Chem.

[39] Case DA, Cheatham TE, Darden T, Gohlke H, Luo R (2005). The Amber biomolecular simulation programs.. J Comput Chem.

[40] Cornell WD, Cieplak P, Bayly CI, Gould IR, Merz KM (1995). A Second Generation Force Field for the Simulation of Proteins, Nucleic Acids, and Organic Molecules.. J Am Chem Soc.

[41] Darden T, York D, Pedersen L (1993). Particle mesh Ewald: An *N*·log(*N*) method for Ewald sums in large systems.. J Chem Phys.

[42] Ryckaert JP, Ciccotti G, Berendsen JC (1977). Numerical integration of the cartesian equations of motion of a system with constraints: molecular dynamics of *n*-alkanes.. J Comput Phys.

[43] Berendsen HJC, Postma JPM, van Gunsteren WF, DiNola A, Haak JR (1984). Molecular dynamics with coupling to an external bath.. J Chem Phys.

[44] Dapprich S, Komaromi I, Byun KS, Morokuma K, Frisch MJ (1999). A new ONIOM implementation in Gaussian98. Part I. The calculation of energies, gradients, vibrational frequencies and electric field derivatives.. J Mol Struct: THEOCHEM.

[45] Humbel S, Sieber S, Morokuma K (1996). The IMOMO method: Integration of different levels of molecular orbital approximations for geometry optimization of large systems: Test for *n*-butane conformation and *S_N_*2 reaction: RCl^+^Cl^−^.. J Chem Phys.

[46] Kuno M, Hannongbua S, Morokuma K (2003). Theoretical investigation on nevirapine and HIV-1 reverse transcriptase binding site interaction, based on ONIOM method.. Chem Phys Lett.

[47] Maseras F, Morokuma K (1995). IMOMM: A new integrated ab initio+molecular mechanics geometry optimization scheme of equilibrium structures and transition states.. J Comput Chem.

[48] Svensson M, Humbel S, Froese RDJ, Matsubara T, Sieber S (1996). ONIOM: A Multilayered Integrated MO+MM Method for Geometry Optimizations and Single Point Energy Predictions. A Test for Diels−Alder Reactions and Pt(P(*t*-Bu)_3_)_2_+H_2_ Oxidative Addition.. J Phys Chem.

[49] Svensson M, Humbel S, Morokuma K (1996). Energetics using the single point IMOMO (integrated molecular orbital+molecular orbital) calculations: Choices of computational levels and model system.. J Chem Phys.

[50] Vreven T, Morokuma K (2000). On the application of the IMOMO (integrated molecular orbital+molecular orbital) method.. J Comput Chem.

[51] Vreven T, Mennucci B, da Silva CO, Morokuma K, Tomasi J (2001). The ONIOM-PCM method: Combining the hybrid molecular orbital method and the polarizable continuum model for solvation. Application to the geometry and properties of a merocyanine in solution.. J Chem Phys.

[52] Vreven T, Morokuma K, Farkas O, Schlegel HB, Frisch MJ (2003). Geometry optimization with QM/MM, ONIOM, and other combined methods. I. Microiterations and constraints.. J Comput Chem.

[53] Grimsby J, Sarabu R, Corbett WL, Haynes NE, Bizzarro FT (2003). Allosteric activators of glucokinase: potential role in diabetes therapy.. Science.

