# Supplementary material for: Lys169 of Human Glucokinase Is a Determinant for Glucose Phosphorylation: Implication for the Atomic Mechanism of Glucokinase Catalysis
Source: PLoS One. 2009 Jul 20;4(7):e6304. doi: 10.1371/journal.pone.0006304 (PMC2706991; doi:10.1371/journal.pone.0006304)
Supplement: Table S1 — Hydrogen bonds existing in the GMAG complex model and their occupancies in the 10-ns MD simulation. (0.05 MB DOC) [file pone.0006304.s001.doc]

**Table S1.** Hydrogen bonds existing in the GMAG complex model and their occupancies in the 10-ns MD simulation.

| No. | Hydrogen Bond | Hydrogen Donor | Hydrogen Acceptor | Existence in Homology Modeling | Occupancy in 10ns-MD simulation of GMAG (%) |
| --- | --- | --- | --- | --- | --- |
| 1 | GK-ATP | Thr82:N | ATP:O^α2^ | Yes | 99.68 |
|  | | Asn83:N | ATP:O^α2^ | Yes | 99.70 |
|  |  | Lys169: N^ζ^ | ATP:O^γ1^ | Yes | 100 |
|  |  | Lys169: N^ζ^ | ATP:O^γ2^ | Yes | 100 |
|  |  | Thr228:O^γ^ | ATP:O^α3^ | Yes | 98.28 |
|  |  | Thr228:O^γ^ | ATP:O^β3^ | Yes | 99.95 |
|  |  | Gly229:N | ATP:O^γ2^ | Yes | 99.97 |
|  |  | ATP:N^1^ | Ser336:O^γ^ | Yes | 99.94 |
|  |  | Ser411:O^γ^ | ATP:O^β2^ | Yes | 99.98 |
| 2 | GK-Glucose | Glucose:O^4^ | Asn204:N^δ1^ | Yes | 99.62 |
|  | | Glucose:O^4^ | Asp205:O^δ1^ | Yes | 97.4 |
|  |  | Glucose:O^3^ | Phe152:N | Yes | 76.26 |
|  |  | Glucose:O^3^ | Glu256:O^ε1^ | Yes | 98.52 |
|  |  | Glucose:O^3^ | Glu256:O^ε2^ | Yes | 100 |
|  |  | Glucose:O^5^ | Lys169:N^ζ^ | Yes | 99.98 |
|  |  | Glucose:O^1^ | Glu290:O^ε1^ | No | 86.46 |
| 3 | ATP-Glucose | Glucose:O^6^ | ATP:O^γ2^ | Yes | 99.67 |
