# Supplementary material for: Lys169 of Human Glucokinase Is a Determinant for Glucose Phosphorylation: Implication for the Atomic Mechanism of Glucokinase Catalysis
Source: PLoS One. 2009 Jul 20;4(7):e6304. doi: 10.1371/journal.pone.0006304 (PMC2706991; doi:10.1371/journal.pone.0006304)
Supplement: Table S2 — Predicted binding free energies of GK and GKK169A mutant with glucose and ATP. (0.03 MB DOC) [file pone.0006304.s002.doc]

**Table S2.** Predicted binding free energies of GK and GK_K169A_ mutant with glucose and ATP *.

|  | Simulation Time (ns) | Glucose (kcal/mol) | ATP(kcal/mol) |
| --- | --- | --- | --- |
| GMAG | 10 | -18.67±4.59 | -44.06±3.94 |
| GK_K169A_- Glucose | 10 | 8.43±2.40 | None |
| GK_K169A_-Mg^2+^-ATP | 10 | None | 25.36±16.45 |
| * The binding free energy was calculated using 100 snapshots from each trajectory when wRMSDs of backbone atoms in each was converged. | | | |
