# Supplementary figures and images for: Lys169 of Human Glucokinase Is a Determinant for Glucose Phosphorylation: Implication for the Atomic Mechanism of Glucokinase Catalysis
Source: PLoS One. 2009 Jul 20;4(7):e6304. doi: 10.1371/journal.pone.0006304 (PMC2706991; doi:10.1371/journal.pone.0006304)

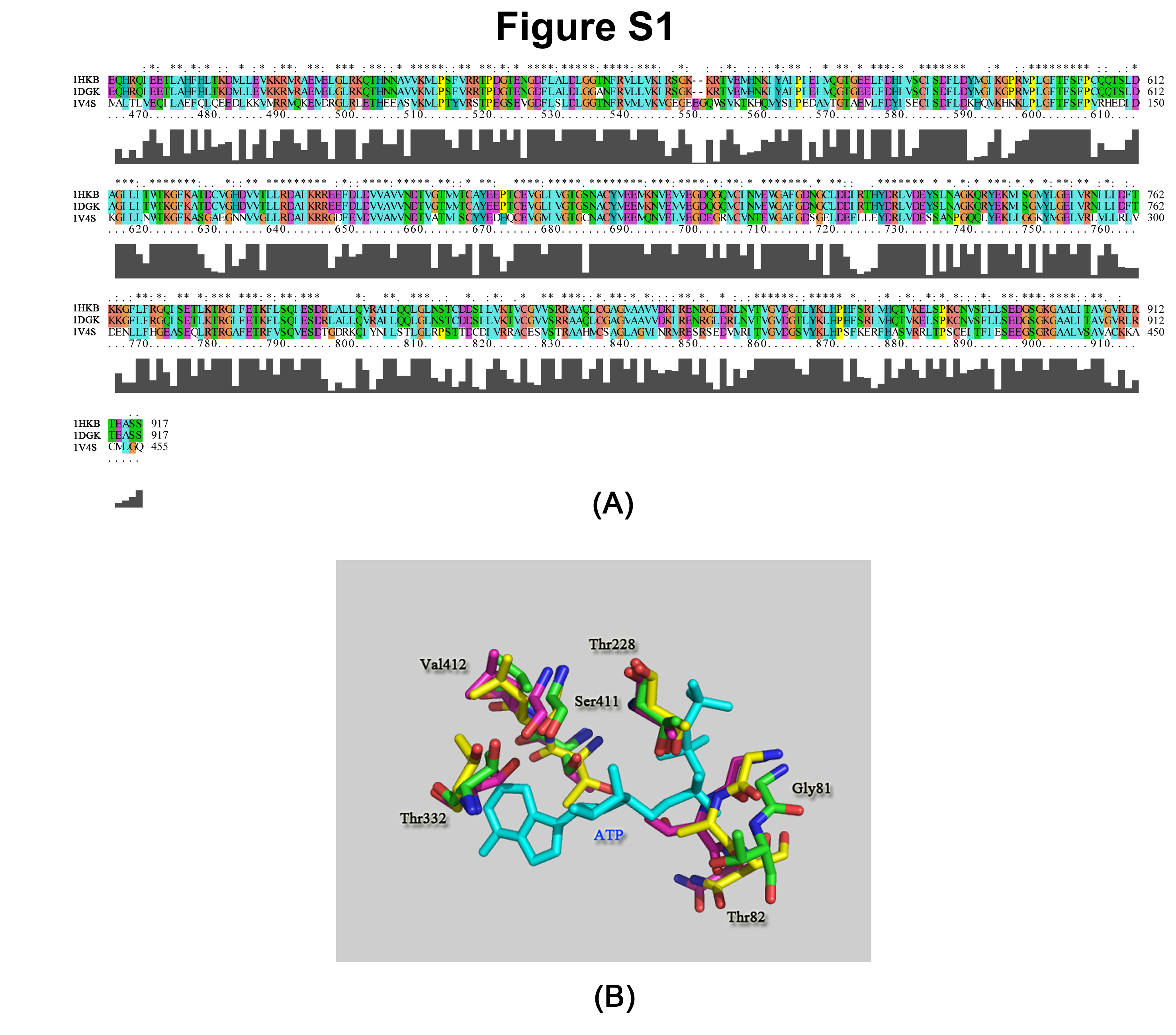

Supplement: Figure S1 — Comparison between GK and homology hexokinases I. (A) The sequence alignment of GK and hexokinases I. (B) Local conformation of residues around ATP binding pocket in the superimposed crystal structures 1DGK(hexokinases I), 1HKC (hexokinases I) and 1V4S(GK). The ATP is colored by cyan. The carbons in 1DGK, 1HKC and 1V4S are colored by yellow, pink and green, respectively. (4.35 MB TIF) [file pone.0006304.s003.tif]

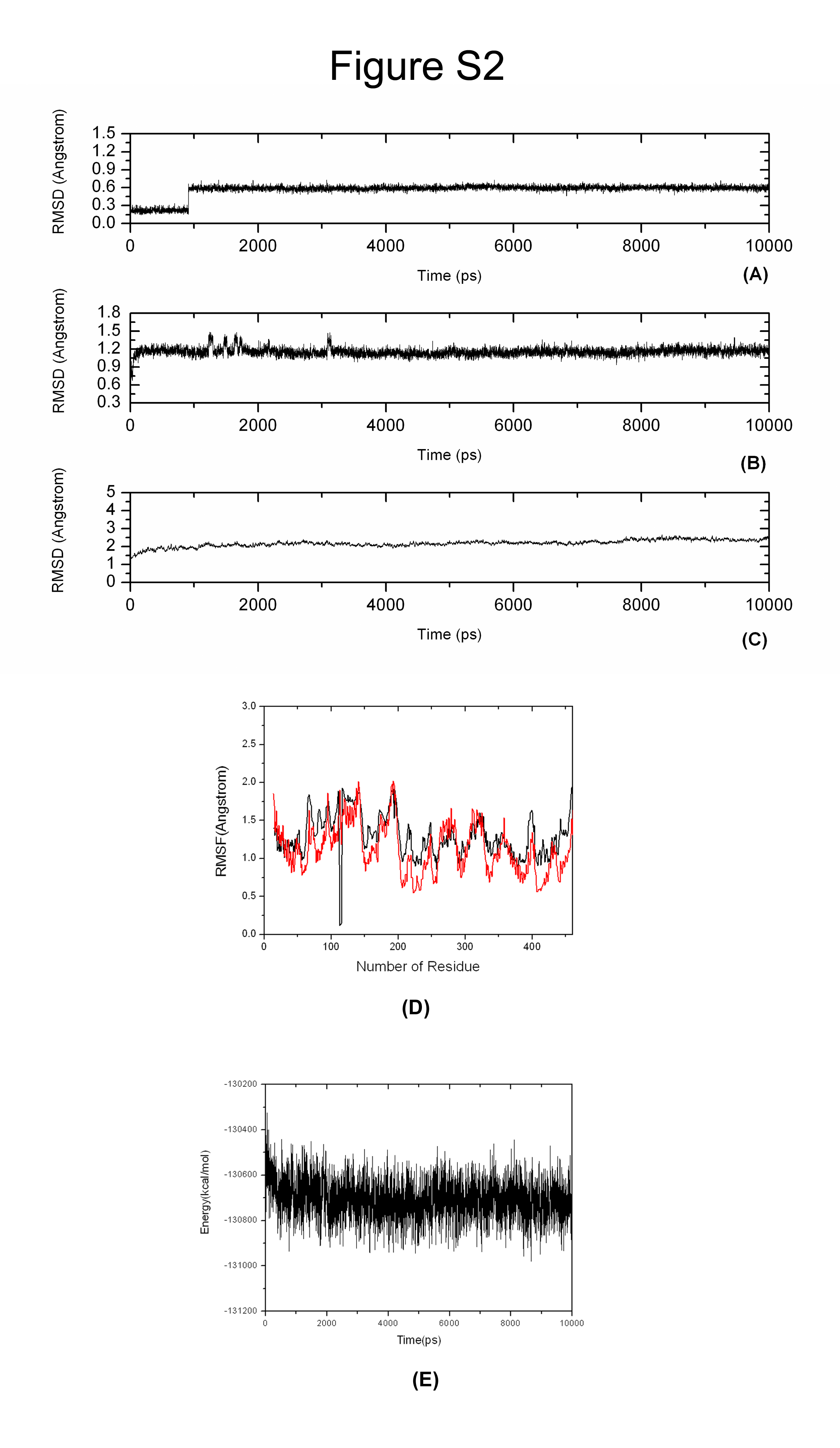

Supplement: Figure S2 — Time dependencies of the weighted Root-Mean-Square Deviations (wRMSDs) for the atoms of glucose (A), ATP (B) and GK (C) from their initial positions during the 10-ns MD simulation. (D) Residue fluctuations obtained by averaging atomic fluctuations over the MD simulation (black curve) and by computing the value from experimentally derived B factors (red curve) for GK crystal structure. (E) Time dependencies of energy during the 10-ns MD simulation. (1.89 MB TIF) [file pone.0006304.s004.tif]

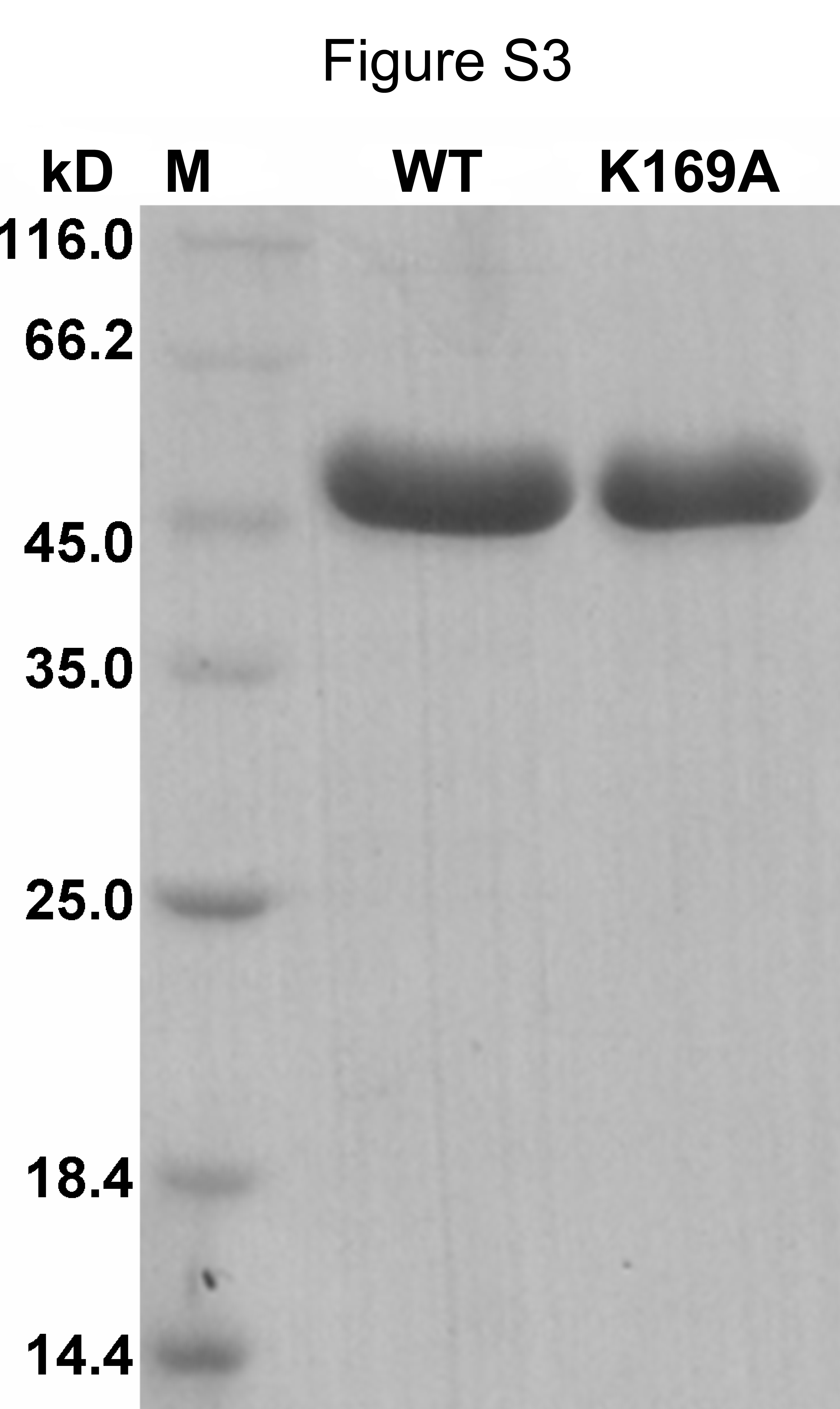

Supplement: Figure S3 — SDS-PAGE for the purified wild-type hLGK2 and its K169A mutant proteins (M: Marker; WT: wild-type hLGK2; K169A: hLGK2 K169A mutant). The molecular weight of wild-type hLGK2 and hLGK2 K169 mutant proteins were evaluated as about 52 kDa indicating by the standard protein markers, which is in agreement with the calculated mass (52 kDa). (8.41 MB TIF) [file pone.0006304.s005.tif]

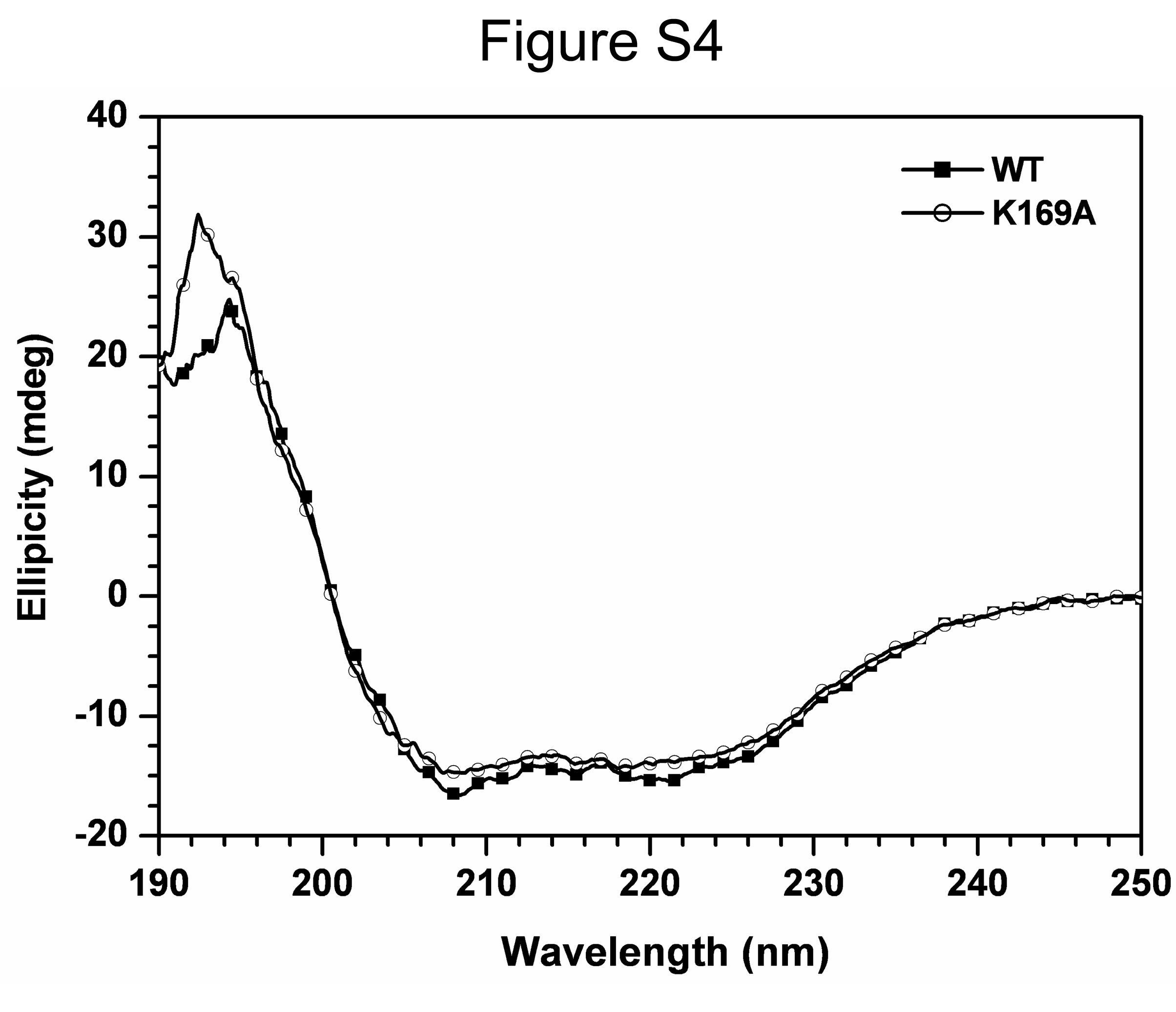

Supplement: Figure S4 — CD spectra of recombinant wild-type hLGK2 and K169A mutant proteins. Far-UV CD spectra of recombinant proteins were monitored at the concentration of 5 µM/L in 20 mM NaH2PO4 buffer (pH 7.1). (0.66 MB TIF) [file pone.0006304.s006.tif]

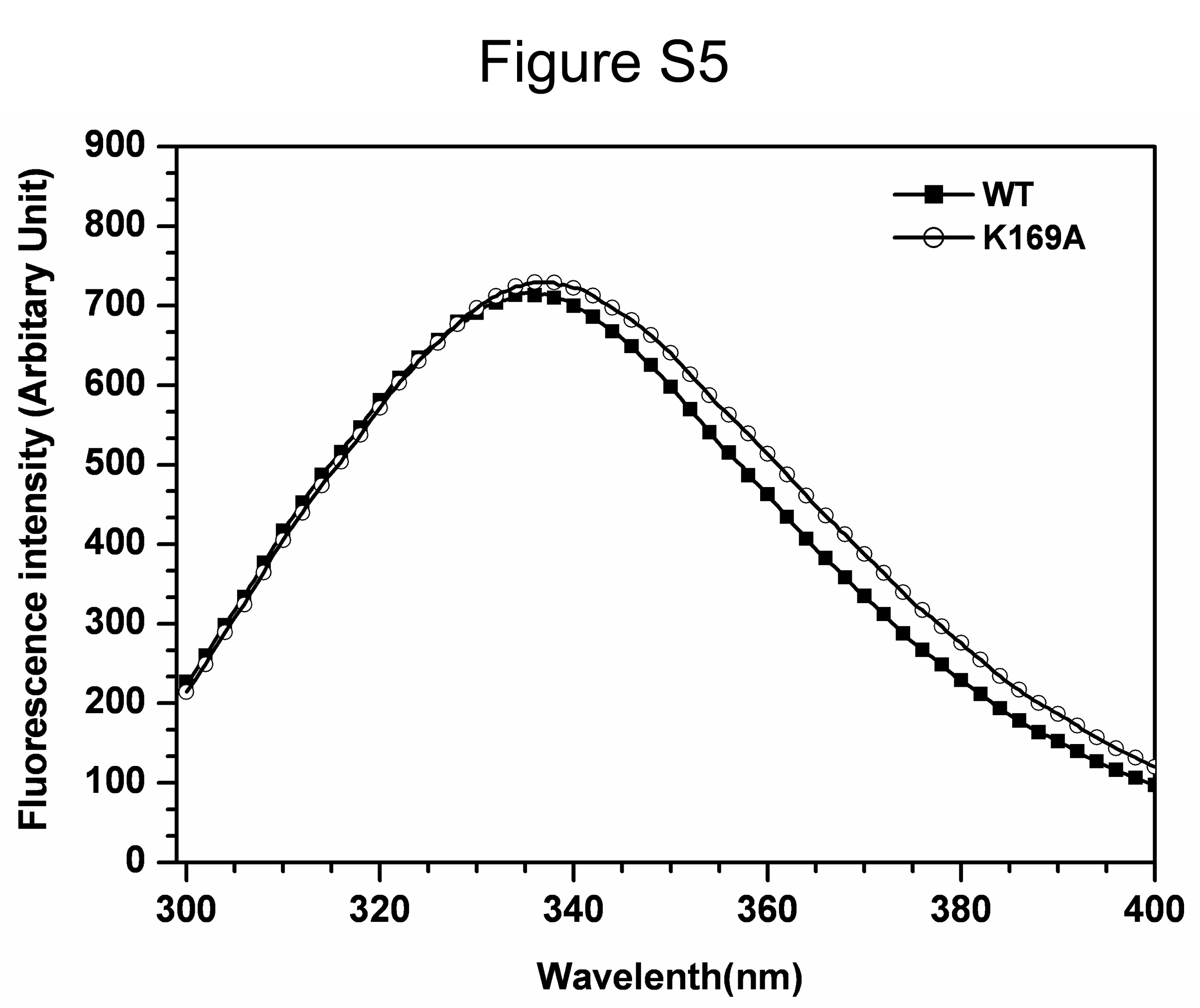

Supplement: Figure S5 — Fluorescence emission spectra of recombinant hLGK2 (wild-type) and K169A mutant proteins. Fluorescence emission spectra of the recombinant proteins were monitored at the concentration of 5 µM/L in 20 mM NaH2PO4 buffer (pH 7.1). (4.27 MB TIF) [file pone.0006304.s007.tif]

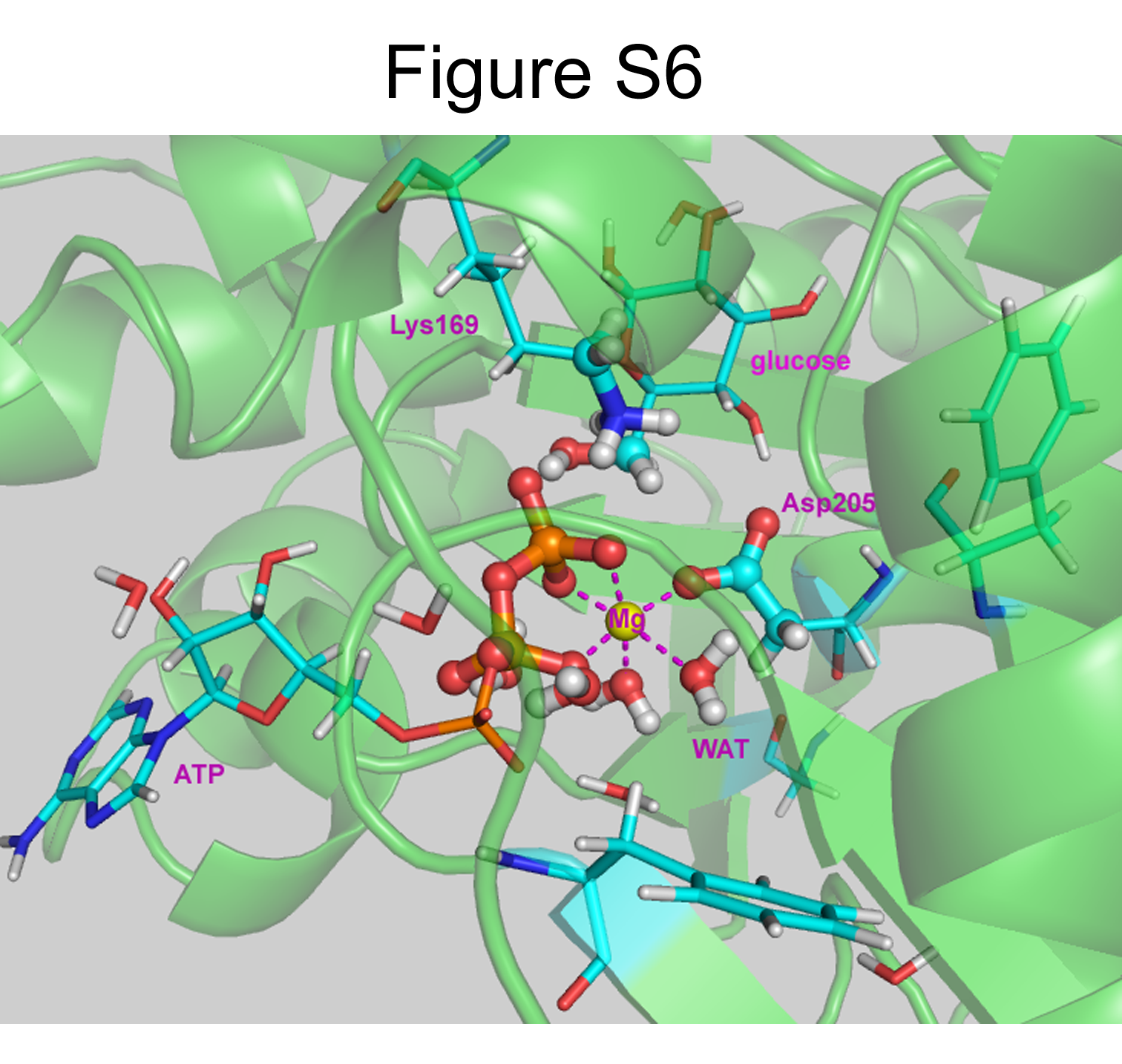

Supplement: Figure S6 — The partitioning scheme of QM and MM regions for the GMAG complex in the QM/MM calculations. Atoms in the QM region are displayed in ball and stick. ATP, Mg2+, glucose and important residues in GK are labeled. (4.45 MB TIF) [file pone.0006304.s008.tif]

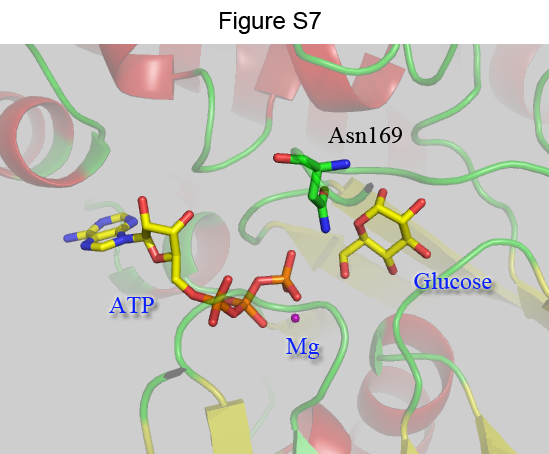

Supplement: Figure S7 — A close view of the configuration of K169N in the environment of GK active site. (0.62 MB TIF) [file pone.0006304.s009.tif]
